# Why compromise the patella? Five-year follow-up results of medial patellofemoral ligament reconstruction with soft tissue patellar fixation

**DOI:** 10.1007/s00264-020-04922-1

**Published:** 2021-01-02

**Authors:** Sebastian P. Boelch, Anna Gurok, Fabian Gilbert, Manuel Weißenberger, Maximilian Rudert, Thomas Barthel, Stephan Reppenhagen

**Affiliations:** 1 Department of Orthopaedic Surgery, University of Wuerzburg, Koenig-Ludwig-Haus, Wuerzburg, Germany; 2 Department of Trauma, Hand, Plastic, and Reconstructive Surgery, University of Wuerzburg, Wuerzburg, Germany

**Keywords:** MPFL, Medial patellofemoral ligament, Patella instability, Patella dislocation, Trochlear dysplasia, Patella alta

## Abstract

**Purpose:**

This study investigates the redislocation rate and functional outcome at a minimum follow-up of five years after medial patellofemoral ligament (MPFL) reconstruction with soft tissue patellar fixation for patella instability.

**Methods:**

Patients were retrospectively identified and knees were evaluated for trochlea dysplasia according to Dejour, for presence of patella alta and for presence of cartilage lesion at surgery. At a minimum follow-up of five years, information about an incident of redislocation was obtained. Kujala, Lysholm, and Tegner questionnaires as well as range of motion were used to measure functional outcome.

**Results:**

Eighty-nine knees were included. Follow-up rate for redislocation was 79.8% and for functional outcome 58.4%. After a mean follow-up of 5.8 years, the redislocation rate was 5.6%. There was significant improvement of the Kujala score (68.8 to 88.2, *p* = 0.000) and of the Lysholm score (71.3 to 88.4, *p* = 0.000). Range of motion at follow-up was 149.0° (115–165). 77.5% of the knees had patella alta and 52.9% trochlear dysplasia types B, C, or D. Patellar cartilage legions were present in 54.2%. Redislocations occurred in knees with trochlear dysplasia type C in combination with patella alta.

**Conclusion:**

MPFL reconstruction with soft tissue patellar fixation leads to significant improvement of knee function and low midterm redislocation rate. Patients with high-grade trochlear dysplasia should be considered for additional osseous correction.

## Introduction

Lateral patella dislocation occurs in 5.8 to 77.8 of 100,000 patients per year [1, 2]. Without surgical treatment redislocation occurs in up to 40–70% [3, 4]. Lateral patella instability can lead to severe functional impairment of the affected knee and recurrent dislocation increases the risk of patellofemoral osteoarthritis [5, 6].

Dejour et al. described trochlea dysplasia (TD) as the pathognomonic feature of patella instability [7]. The biomechanical study by Senavongse and Amis described the complex interaction of the patella stabilizing structures such as the musculus vastus medialis obliquus, the trochlea, and the medial patellofemoral ligament (MPFL) [8]. Next to TD, patella alta quantified by the Caton-Deschamps Index (CDI) correlates with the manifestation of patella instability [7]. Increased tuberositas tibiae-tibial groove (TT-TG) distance is another crucial risk factor for patellar instability, which is another means of quantification of patellofemoral alignment [9].

With some restrictions, treatment algorithms advised conservative treatment for first patella dislocation in the past [3, 10]. However, rupture of the MPFL, which typically comes along with first patella dislocation, is considered a major cause for the development of persisting lateral patella instability and recurrent dislocations [11, 12]. MPFL reconstruction improves knee function and reduces the risk of redislocation [13, 14]. Based on this experience, surgical treatment for patients with predisposing factors of lateral patella instability is increasingly recommended even before redislocation occurs [15]. MPFL reconstruction with autologous tendon grafts therefore has evolved to the standard of care for the treatment of lateral patellar instability [3, 10, 14, 16].

Several techniques for bony fixation of the tendon graft to the patellar insertion site have been described. These techniques require either patellar drilling or bone anchor systems [17]. Patella fracture, foreign body irritation, and implant failure are clinical relevant complications observed with these bony fixation techniques [13, 17, 18].

A soft tissue fixation technique with resorbable sutures, which does not compromise the patellar bony integrity, is favored at the study institution for MPFL reconstruction. With this approach, significant improvement of knee function and a redislocation rate of 3.7% after one year were demonstrated in 2015 [19]. However, to date, it has not been investigated whether this technique provides sufficient patella stability over midterm.

It was hypothesized that the redislocation rate for MPFL reconstruction with soft tissue patellar fixation is comparable with the results of osseous patellar fixation described in the literature. It was also investigated whether there is significant functional improvement after a minimum five year follow-up.

## Methods

### Patient inclusion and exclusion

This study was approved by the institution’s ethics review board (IRB-Number 147/16-ge). The electronic database of the study institution was retrospectively scanned for patella dislocations treated with MPFL reconstruction with an autologous gracilis tendon graft and soft tissue patellar fixation performed between 2010 and 2012. In patients with significant misalignment, such as a TT-TG > 20 mm, MPFL reconstruction was combined with tuberosity transfer. Since the aim was to investigate the results after isolated MPFL reconstruction, these patients were excluded. Further exclusion criteria were syndromal underlying diseases, previous total knee arthroplasty on the affected knee, and indication for total knee arthroplasty before follow-up.

### Surgical treatment

**Fig. 1:**
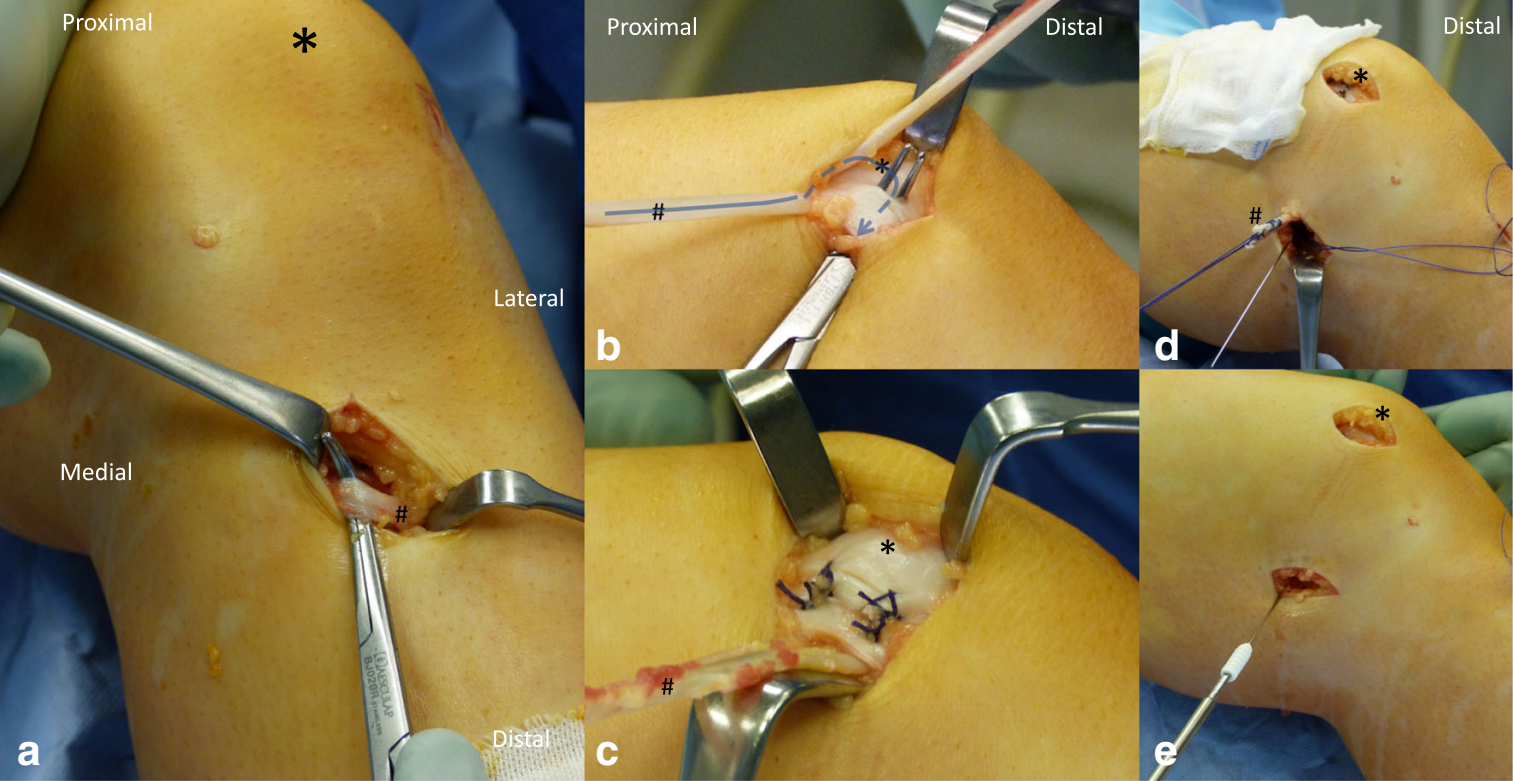
Intra-operative images of MPFL reconstruction with soft tissue patellar fixation on a left knee. *Indication of the position of the patella; ^#^the musculus gracilis tendon graft. **a** Harvest of the tendon graft at the pes anserinus. **b** Proximally, the tendon graft was weaved through the soft tissue around the patella. The distal end of the graft is going to be weaved back again in a u shape as indicated by the arrow. **c** Fixation with resorbable sutures. **d** The graft is passed within the fascial layers dorsally. **e** Fixed to the anatomical femoral insertion site of the MPFL with a resorbable interfering screw.

Arthroscopy was performed simultaneously before MPFL reconstruction. A musculus gracilis tendon autograft (Fig. 1a) was transferred through the medial capsule in the anatomical layer of the MPFL (Fig. 1b) and weaved in a u shape through the capsule and the periosteum of the patella. Resorbable suture material was used for fixation (Vicryl USP 0, Ethicon Johnson & Johnson, USA) (Fig. 1c). At the femoral anatomical insertion site, the graft was fixed with a resorbable interference screw (Fig. 1d and e). The procedure has been published in detail before [19].

### Parameters assessed

**Fig. 2:**
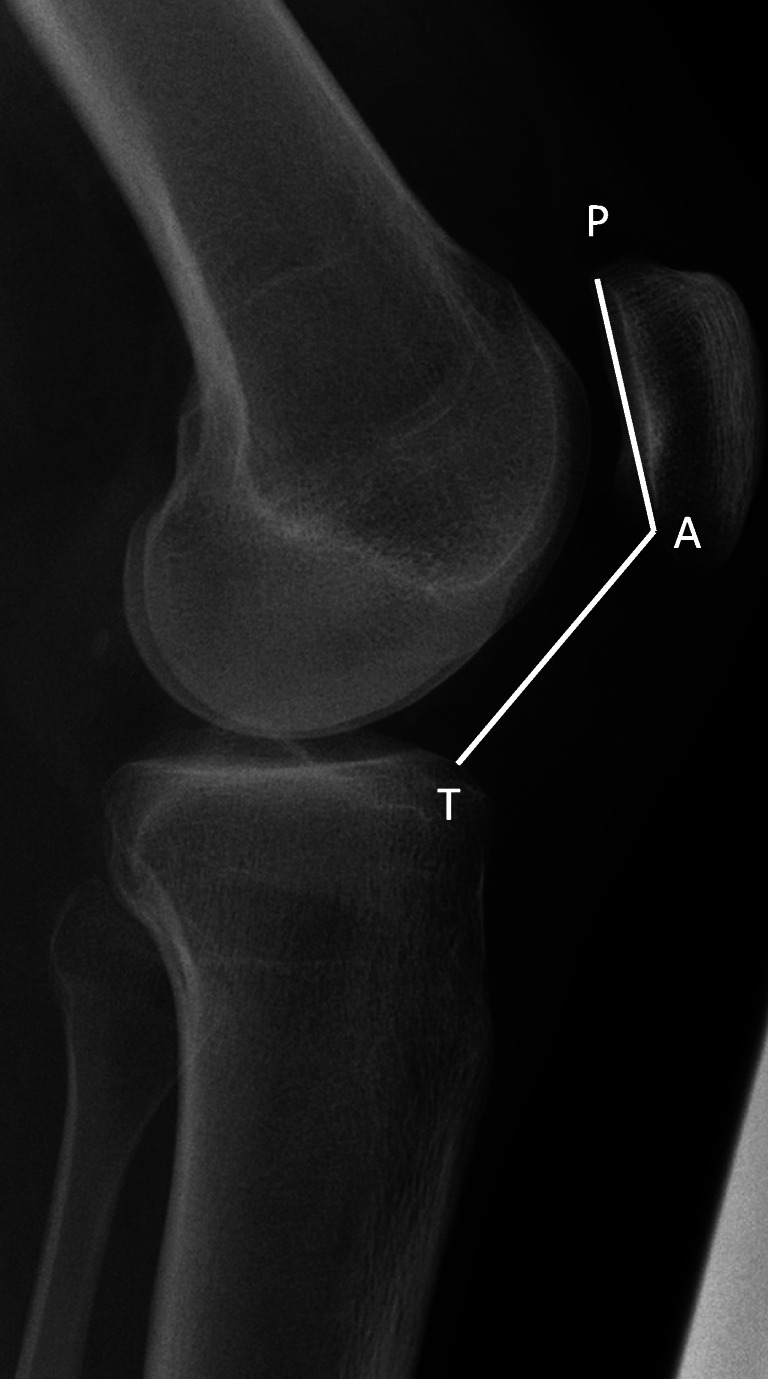
Calculation of the Caton-Dechamps Index on lateral radiographs with the knee in 20–30° flexion showing patella alta: the ratio of distance P to A and distance A to T is > 1.2; P and A, superior and inferior margins of the patellar articular surface; T, anterior tibial plateau

**Fig. 3:**
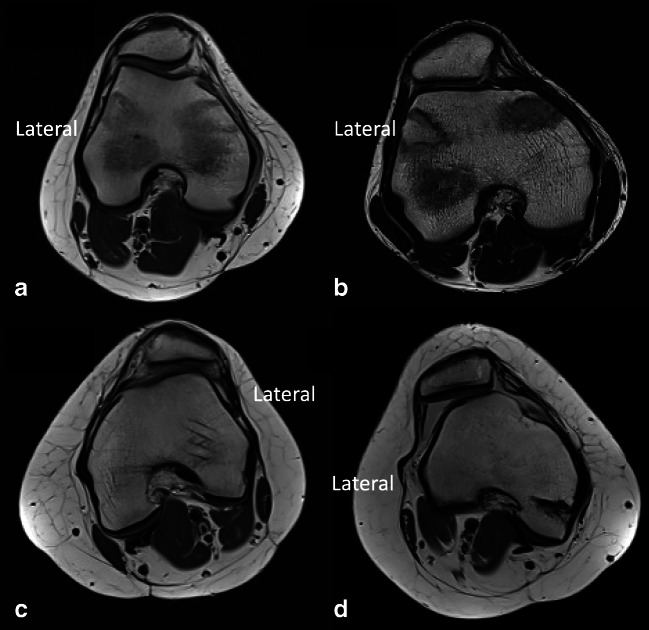
Examples of trochlea dysplasia evaluated on axial MRI. Evaluation is performed on the most proximal axial image with the complete cartilaginous trochlea. **a** Type A in a right knee with a shallow trochlea. **b** Type B in a right knee with a flat to convex trochlea. **c** Type C in a left knee with asymmetry of the trochlear facets and hypoplastic medial condyle. **d** Type D in a right knee with asymmetry of the trochlear facets and the medial facet appears elevated. Note that the patella is laterally dislocated.

For patients’ characteristics, history of previous operations on the affected knee was extracted from the patient charts. Presence of cartilage lesion with description of location and classification according to Outerbridge was tabulated from the surgical reports at MPFL reconstruction [20]. CDI and the type of TD according to Dejour et al. were evaluated on preoperative radiographs and MRIs [7]. The methods are depicted in Figs. 2 and 3.

For outcome, patients were invited for follow-up examination between January and December 2017. At this follow-up, patients completed the questionnaires on the current and pre-operative Kujala, Lysholm, and Tegner score. Range of motion was measured. The patients were asked about subsequent surgeries and events of redislocations. Patients who failed to appear for examination were contacted by telephone.

### Statistics

Parameters are depicted by mean and range. For the reference redislocation rate of osseous patellar graft fixation reported in the literature, we referred to the recent review article by Song et al. [21]. Pre-operative to follow-up scores were compared with the Wilcoxon signed-rank test. Correlation of categorical parameters was calculated with Cramer’s *V* and of ordinal with metric parameters with Spearman rank correlation. Only sample sizes with *N* > 5 were tested. *p* was set significant at < 0.05. Statistics were conducted with SPSS 23 (SPSS Inc. USA).

## Results

**Fig. 4:**
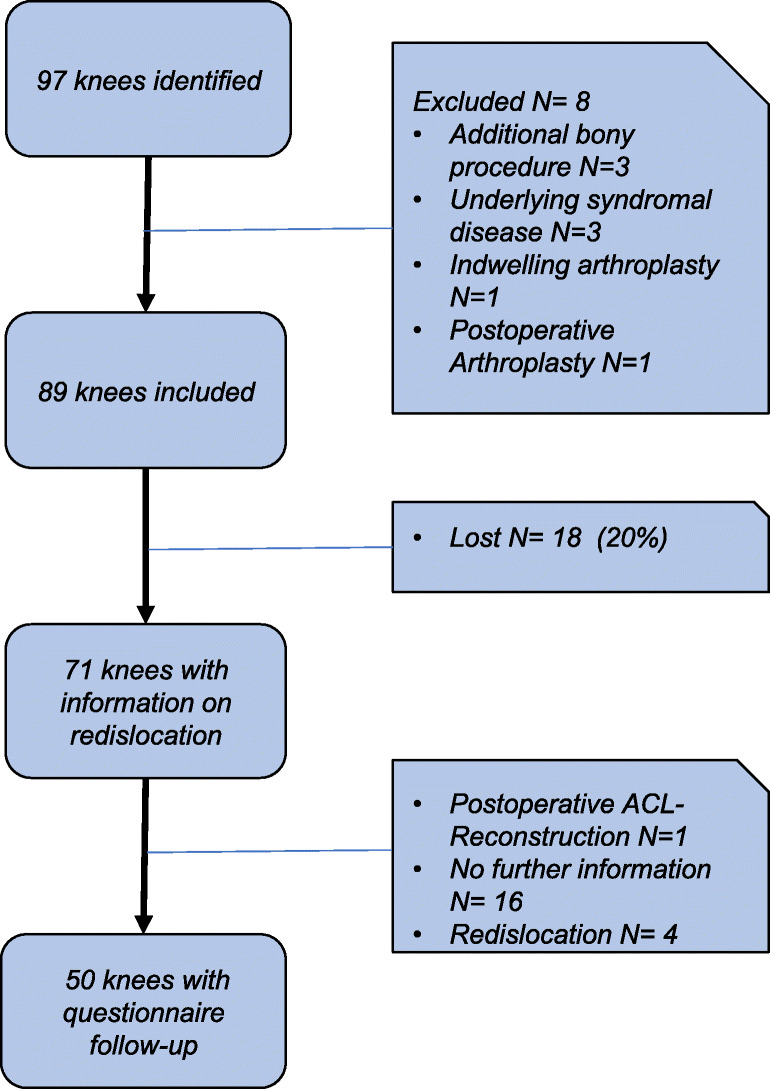
Patient inclusion; ACL anterior cruciate ligament

Eighty-nine patients were included. Information on post-operative redislocation was obtained for 71 of 89 knees (79.8%) in 68 patients, which represent the investigated population. Follow-up questionnaires were received for 50 knees (70.4%) (Fig. 4).

38.0% were knees of male patients. Mean age at surgery was 20.9 years (10–47). Growth plates were closed in 76.1%. The left knee was affected in 47.9%. 77.5% knees had no history of previous surgery. 18.3% had previous medial reefing and lateral release. 4.2% of knees had other previous operations: one removal of a chondral flake, one refixation, and the other reconstruction of the anterior cruciate ligament and partial resection of the lateral meniscus. Patellar cartilage lesions were present in 54.2%: of these, 23.1% with grade 1, 35.6% with grade 2, 28.3% with grade 3, and 13.0% with grade 4. 19.1% of knees were classified to have a normal trochlea, type A TD was observed in 27.9 %, type B was observed in 29.4 %, type C was observed in 19.1%, and 4.4% had TD type D. Another three knees could not be classified (4.4 %). Mean CDI was 1.4 [1, 2]. In 77.5 % of the knees, patella alta (CDI > 1.2) was observed (Table 1).

**Table 1:** Trochlea dysplasia (TD) grouped by Caton-Deschamps Index (CDI)

TD type	Total *n* (%)	CDI ≤ 1.2 *n* (%)	CDI > 1.2 *n* (%)
Normal	13 (19.1)	5 (6.9)	8 (11.1)
A	19 (27.9)	2 (2.8)	17 (11.1)
B	20 (29.4)	6 (8.3)	14 (19.4 )
C	13 (19.1)	1 (1.4)	12 (16.6)
D	3 (4.4)	0 (0.0)	3 (4.4)
n.c.	3 (4.4)	/ *	1 (1.4)
Total	71 (100)	14* (19.7*)	55 (77.5)

*n.c.* not classified; *2 knees (2.8% of total) could neither for CDI nor for TD be classified

19.7% (*N* = 14) of knees were treated after first patella dislocation and all others after recurrent patella dislocation. After a mean follow-up of 5.8 years (69.8 months (59–86)), the redislocation rate was 5.6%. Redislocations occurred within 28 months after the procedure. All four occurred exclusively in the 13 knees with TD type C knees with CDI *>* 1.2. The correlation was highly significant (Cramer’s *V* 0.542, *p* = 0.000).

**Fig. 5:**
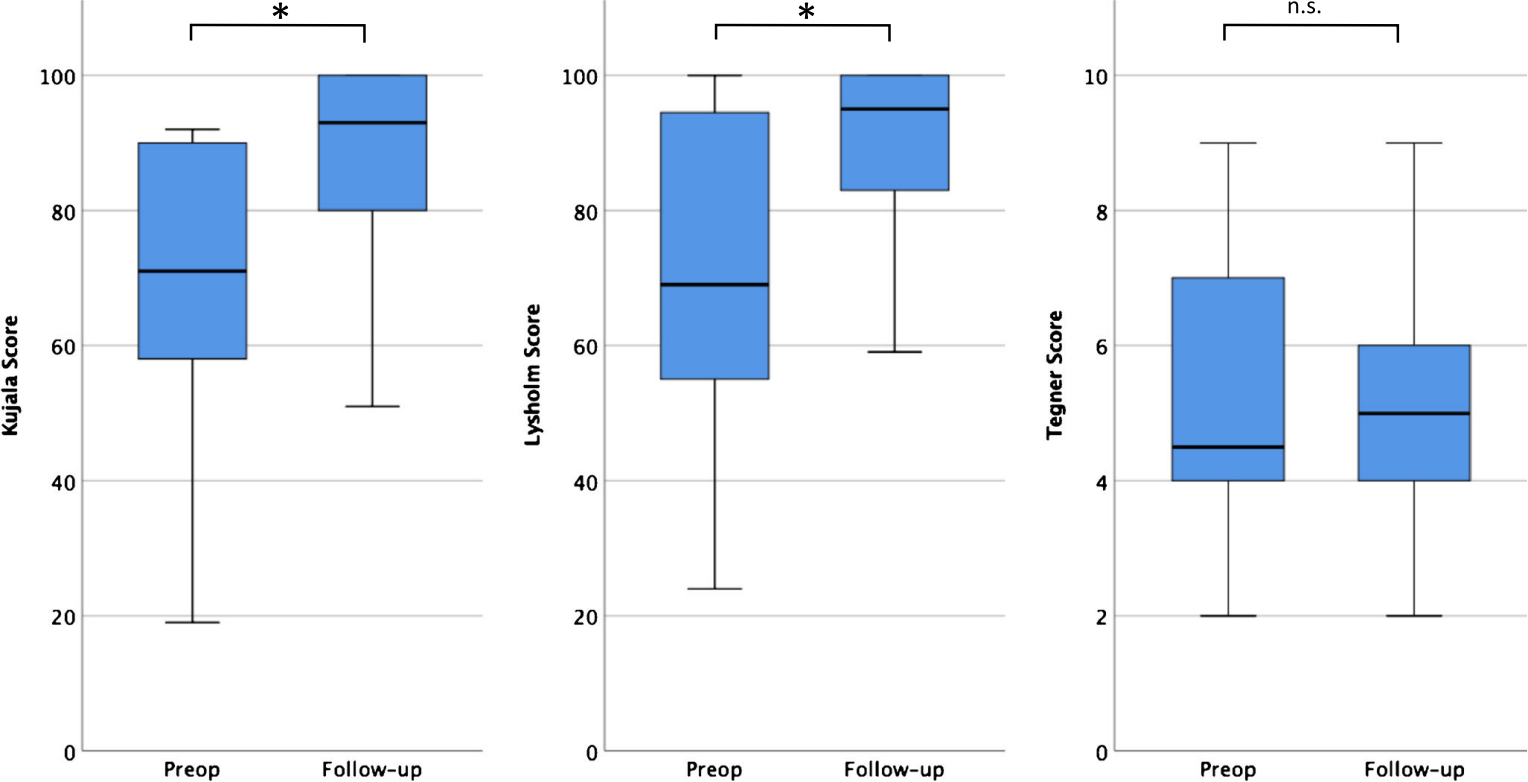
Functional scores (Kujala, Lysholm, and Tegner score) preoperative and at 5.8-year follow-up (range 59–86 months); *p < 0.05; n.s. not significant

The Kujala and the Lysholm score improved significantly from 68.8 (19–92) (*N* = 47) and 71.3 (24–100) (*N* = 48) before MPFL reconstruction to 88.2 (49–100) (*N* = 47) (*p* = 0.000) and 88.4 (28–100) (*N* = 48) (*p* = 0.000) at follow-up. Tegner score was already low at the time of surgery (5.12 (2–9) (*N* = 50) and did not significantly change to follow-up (5.0 (2–9) (*N* = 50) (*p* = 0.497)) (Fig. 5). Range of motion at follow-up was 149.0° (115–165).

Stratification of TD to mild (normal or type A) or to high (types B, C, and D) did not affect the significant improvement in Kujala and Lysholm score. The same was observed for stratification by selected patient factors such as CDI and grading of patellar cartilage lesions. Improvement was observed in all subgroups, however, not always significant. Table 2 depicts these changes from pre-operative to follow-up for the Kujala score and Table 3 for the Lysholm score. Factors that did affect pre-operative functional score were the numbers of pre-operative dislocations and a history of preceding surgery for patellar instability. Spearman correlation for a number of pre-operative dislocations was *ρ* − 0.4 for pre-operative Kujala score (*p* = 0.003) and *ρ* − 0.3 for pre-operative Lysholm score (*p* = 0.038). Pos-toperative functional scores did not correlate significantly (*p* = 0.948 and 0.750). For a history of preceding surgery for patella instability before MPFL reconstruction, Spearman correlation was *ρ* 0.4 (*p* = 0.014) for pre-operative Kujala and *ρ* 0.2 (*p* = 0.221) for pre-operative Lysholm score. This factor, a history of surgery for patella instability, significantly correlated with the Lysholm at follow-up score (*ρ* − 0.4; *p* = 0.008), as well as with the Kujala score at follow-up (*ρ* − 0.4; *p* = 0.005).

**Table 2:** Comparison of Kujala score pre-operatively and at follow-up stratified to patient factors

		Pre-operatively Mean (range)	Follow-up Mean (range)	*p*
Trochlear dysplasia	Mild (*n* = 20)	71.8 (30–92)	88.1 (52–100)	*0.003*
	High (*n* = 25)	67.48 (19–92)	88.24 (49–100)	*0.000*
*p*		0.638	0.863	
CDI	≤ 1.2 (*n* = 10)	66.1 (34–92)	92.3 (51–100)	*0.007*
	> 1.2 (*n* = 36)	70.25(19–92)	87.33(49–100)	*0.000*
*p*		0.566	0.165	
Number of pre-operative dislocations	1 (*n* = 8)	81.9 (19–92)	94.8 (86–100)	0.071
	2 (*n* = 9)	74.2 (32–92)	85.1 (52–100)	0.236
	≥ 3 (*n* = 29)	63.34 (30–90)	87.8 (49–100)	*0.000*
*p* (1 vs > 1)		*0.006*	0.613	
Preceding surgery for pat instability	no (*n* = 36)	72.1 (19–92)	91.5 (52–100)	*0.000*
	yes (*n* = 10)	57.3 (30–79)	77.2 (49–98)	*0.011*
*p*		*0.016*	*0.005*	
Grading of patellar cartilage lesions	< 3 (*n* = 30)	67.7 (30–92)	87.9 (51–100)	*0.000*
	≥ 3 (*n* = 16)	71.5 (19–92)	88.6 (49–100)	*0.001*
*p*		0.470	0.991	

Trochlear dysplasia was graded according to Dejour and stratified to mild in case of no or type A and to high in case of types B, C, or D; *CDI* Caton-Deschmaps Index; cartilage lesions were graded according to Outerbridge; italics indicate significance

**Table 3:** Comparison of Lysholm score pre-operatively and at follow-up stratified to patient factors

		Pre-op Mean (range)	Follow-up Mean (range)	*p*
Trochlear dysplasia	Mild (*n* = 21)	73.6 (30–100)	88.2 (49–100)	*0.013*
	High (*n* = 25)	71.3 (24–100)	87.8 (28–100)	*0.009*
*p*		0.731	0.982	
CDI	≤ 1.2 (*n* = 10)	69.1 (30–100)	89.8 (28–100)	0.093
	> 1.2 (*n* = 37)	73.1 (24–100)	87.86 (49–100)	*0.001*
*p*		0.592	0.337	
Number of pre-op dislocations	1 (*n* = 9)	89.3 (24–100)	96.1 (84–100)	0.715
	2 (*n* = 9)	73 (34–100)	83.9 (49–100)	0.312
	≥ 3 (*n* = 30)	66.4 (29–100)	88.0 (28–100)	*0.000*
*p* (1 vs >1)		*0.016*	0.199	
Preceding surgery for pat. Instability	No (*n* = 36)	73.9 (24–100)	91.8 (49–100)	*0,000*
	Yes (*n* = 11)	64.7 (36–87)	77.73 (28–100)	0.100
*p*		0.180	*0.008*	
Grading of patellar cartilage lesion	< 3 (*n* = 30)	70.9 (29–100)	89.0 (28–100)	*0.002*
	≥ 3 (*n* = 17)	72.0 (24–100)	87.33 (60–100)	*0.011*
*p*		0.830	0.616	

Trochlear dysplasia was graded according to Dejour and stratified to mild in case of no or type A and to high in case of types B, C, or D; *CDI* Caton-Deschamps Index; cartilage lesions were graded according to Outerbridge; italics indicate significance

The TD type C with patella alta subgroup did not significantly improve in clinical scores with *p* = 0.144, *p* = 0.255, and *p* = 0.180 for Kujala, Lysholm, and Tegner score, respectively.

## Discussion

Isolated MPFL reconstruction resulted in a low post-operative redislocation rate of 5.6% and significant clinical improvement of the knees’ function and patient’s perception. MPFL reconstruction restores the primary stabilization of the patella [11, 12] and is the standard of care for the treatment of lateral patella instability [21]. Osseous fixation techniques of the graft at the patella have been described with suture anchors, interference screws, and different drilling methods. These osseous fixation techniques are associated with the risk of implant failure and patella fracture [13, 17]. Although only sparsely discussed in the literature, Hopper et al. reported a patella fracture rate of 5.6% with an interference screw technique [18]. In contrast to these techniques, the investigated soft tissue fixation does not compromise the bone stock of the patella. Instead, the graft is weaved in a u shape through the capsule and the periosteum of the patella and fixed with resorbable sutures. For this quick and easy technique, the current study could demonstrate a low redislocation rate comparable to the one reported for osseous fixation techniques in the literature by Song et al. [21]. While for their review focusing on MPFL reconstruction, knees with TD or patella alta were excluded, in the current study, these knees were included and treated merely with isolated MPFL reconstruction. Recent treatment recommendations increasingly advise combining MPFL reconstruction with bony correction in case of abnormal osseous anatomy [3]. Rhee et al. specifically emphasized to correct patella alta by transfer of the tuberosity [10]. However, Hopper et al. showed that MPFL reconstruction in combination with tuberosity transfer does not guarantee stable patellae [18]. In the current study, we have not observed redislocations with patella alta, if TD was only mild or not present. By methods, patients with TT-TG larger than 20 mm were excluded from the study. Increased TT-TG displays a significant pathology for lateral patellar instability, which we believe should be treated with additional tuberosity transfer. All redislocations in the current study occurred in knees with severe TD, concurrent patella alta, and a TT-TG < 20 mm. For this subgroup, the post-operative redislocation rate was 13.8%. Balcarek et al. compared two treatment modalities in knees with severe TD but without patella alta: the knees treated with trochleoplasty and MPFL reconstruction had a redislocation rate of 2.1% whereas the rate for the knees treated with MPFL reconstruction only was 7.0%. But this difference was not significant [14]. Still, the authors recommend to consider trochleoplasty as primary treatment option in knees with severe TD [3]. This study comprises three cases with type D TD. No redislocation was seen in this subgroup, but two of the three patients reported positive apprehension sign in clinical examination. Thus, in knees with severe TD and patella alta, additional bony correction with trochleoplasty should be strongly considered.

There are several limitations to this study. Correctly completed questionnaires were obtained from only 67.6% (*n* = 48). The missing questionnaires were caused by two problems. First is inability to complete the questionnaires and to send them to the study institution, if an appointment for clinical examination was not possible. Second is not matching answer possibilities leading to no, multiple, or incorrect answers. However, statistics resulted in highly significant results and the mean values compare closely to those reported in the literature [14, 22]. Further, a recall bias for the questionnaires retrieving the preoperative condition might be relevant. However, the retrospective assessment of the scores certainly implements the patients’ satisfaction with the procedure.

This study investigated subgroups stratified by the Dejour classification system, which is originally based on radiographs. High inter- and intra-observer variability needs to be acknowledged for this qualification of TD. Thus, in 53.5% of the knees, the stratification was cross-checked on axial MRIs, which has proven more reproducible results [23].

Although not particularly investigated in the current study, soft tissue fixation does not require intra-operative fluoroscopy for placement control and might even decrease operation time. The soft tissue fixation technique does not produce a bony defect at the patella, which is an advantage of the described soft tissue patellar fixation technique, and thus, implant- or drilling-associated complications are avoided. As shown in the current study, more than half the knees with patella instability have cartilage lesions and thus, future patellar cartilage reconstructive or replacing therapies impede. Under these considerations, MPFL reconstruction with soft tissue patella fixation is a highly reasonable option that after a follow-up of 5.8 years now has proven very low post-operative redislocation rates and significant improvement in knee function scores.

## Conclusion

MPFL reconstruction with soft tissue patellar fixation leads to very low midterm redislocation rate comparable to the one reported in the literature and to significant improvement of knee function. Patients with high-grade TD should be considered for additional bony correction.

## Data Availability

The datasets used and analyzed during the current study are available from the corresponding author on reasonable request.
